# A simple method for detecting oncofetal chondroitin sulfate glycosaminoglycans in bladder cancer urine

**DOI:** 10.1038/s41420-020-00304-z

**Published:** 2020-07-27

**Authors:** Thomas Mandel Clausen, Gunjan Kumar, Emilie K. Ibsen, Maj S. Ørum-Madsen, Antonio Hurtado-Coll, Tobias Gustavsson, Mette Ø. Agerbæk, Francesco Gatto, Tilman Todenhöfer, Umberto Basso, Margaret A. Knowles, Marta Sanchez-Carbayo, Ali Salanti, Peter C. Black, Mads Daugaard

**Affiliations:** 1 Department of Urologic Sciences, University of British Columbia, Vancouver, BC Canada; 2 Vancouver Prostate Centre, Vancouver, BC Canada; 3 Centre for Medical Parasitology at Department for Immunology and Microbiology, Faculty of Health and Medical Sciences, University of Copenhagen and Department of Infectious Disease, Copenhagen University Hospital, Copenhagen, Denmark; 4 Department of Pathology and Laboratory Medicine, University of British Columbia, Vancouver, BC Canada; 5 VarCT Diagnostics ApS, Copenhagen, Denmark; 6 Department of Biology and Biological Engineering, Chalmers University of Technology, Göteborg, Sweden; 7 Department of Urology, University Hospital Tübingen, Eberhard-Karls University Tübingen, Tübingen, Germany; 8 Studienpraxis Urologie, Clinical Trial Unit, Steinengrabenstr. 17, Nürtingen, Germany; 9 Medical Oncology Unit 1, Istituto Oncologico Veneto IOV – IRCCS, Padova, Italy; 10 Division of Molecular Medicine, Leeds Institute of Medical Research at St James’s, St James’s University Hospital, Beckett Street, Leeds, UK; 11 Health Sciences Department, Universidad Pontificia de Salamanca, Salamanca, Spain; 12 Present Address: Elypta AB, Stockholm, Sweden

**Keywords:** Diagnostic markers, Bladder cancer

## Abstract

Proteoglycans in bladder tumors are modified with a distinct oncofetal chondroitin sulfate (ofCS) glycosaminoglycan that is normally restricted to placental trophoblast cells. This ofCS-modification can be detected in bladder tumors by the malarial VAR2CSA protein, which in malaria pathogenesis mediates adherence of parasite-infected erythrocytes within the placenta. In bladder cancer, proteoglycans are constantly shed into the urine, and therefore have the potential to be used for detection of disease. In this study we investigated whether recombinant VAR2CSA (rVAR2) protein could be used to detect ofCS-modified proteoglycans (ofCSPGs) in the urine of bladder cancer patients as an indication of disease presence. We show that ofCSPGs in bladder cancer urine can be immobilized on cationic nitrocellulose membranes and subsequently probed for ofCS content by rVAR2 protein in a custom-made dot-blot assay. Patients with high-grade bladder tumors displayed a marked increase in urinary ofCSPGs as compared to healthy individuals. Urine ofCSPGs decreased significantly after complete tumor resection compared to matched urine collected preoperatively from patients with bladder cancer. Moreover, ofCSPGs in urine correlated with tumor size of bladder cancer patients. These findings demonstrate that rVAR2 can be utilized in a simple biochemical assay to detect cancer-specific ofCS-modifications in the urine of bladder cancer patients, which may be further developed as a noninvasive approach to detect and monitor the disease.

## Introduction

Bladder cancer is the fifth most common cancer in the world and one of the most expensive cancers to treat on a per-patient basis due to the need for constant surveillance and multiple therapeutic interventions^1,2^. Urinary biomarkers in bladder cancer have been researched for decades with the aim of noninvasive detection of disease and to monitor high-risk patients with nonmuscle invasive bladder cancer (NMIBC)^3–5^. While several urine biomarkers have been identified over the years, novel biomarkers with high sensitivity and specificity are in demand^5^.

Chondroitin Sulfate (CS) is a glycosaminoglycan (GAG) linked to specific proteoglycans (CSPGs) present in the cell membranes, or secreted into the extracellular matrix and bodily fluids^6^. CS chains are comprised of linear polysaccharides made up of repeated N-acetyl-D-galactosamine (GalNAc) and Glucuronic Acid (GlcA) disaccharide units and can vary greatly in length. While the basic structure is simple, an immense structural heterogeneity is achieved through modifications of the carbohydrate backbone, such as sulfation of component hydroxyl groups. The nontemplate driven synthesis ofCS that vary in size and sulfation patterns makes CS amongst the most heterogeneous molecules in the human body^7^.

Bladder cancer, like most other solid tumors, expresses a distinct form of chondroitin sulfate (CS), normally restricted to the placenta^8^. In the placenta, this CS modification plays a key role in the lifecycle of *Plasmodium falciparum* malaria parasites. During malaria infections, the malaria parasite expresses specific host-anchor proteins on the surface of infected erythrocytes, which enables them to adhere to the vascular bed and avoid destruction in the spleen^9^. Depending on the anchor-protein expressed, infected erythrocytes can adhere to different organs in the human body including brain, lung, heart, and placenta^10^. The placenta-specific malaria tropism is mediated by the parasite-encoded host-anchor-protein VAR2CSA, which facilitates specific adherence of infected erythrocytes to placental CS chains with no adhesion to any other tissues in the human body, despite CS being present on many cells of the human host^11^. The strict specificity for CS in the placenta indicates the presence of a unique CS variant structurally distinct from other CS types, and that the malarial VAR2CSA has been evolutionarily optimized for selectivity against this subtype ofCS only^12^. Interestingly, ofCS is also expressed in most types of solid tumors^8^. The re-expression of a fetal antigen is consistent with the idea that cancers revert to a less differentiated (or fetal) state during disease progression to facilitate proliferation, migration, and other oncogenic processes. Because of the similarities between placenta and tumors, recombinant malarial VAR2CSA (rVAR2) can be conveniently utilized to detect ofCS in cancers and facilitate delivery of toxic payloads to tumors in vivo, including cisplatin resistant bladder cancer^8,13^.

Several studies have reported changes in GAG concentration and composition in bodily fluids in a variety of pathologies, including cancer^14–20^. In fact, changes in the urine GAG composition have been previously suggested as a biomarker for detection of cancers, including ovarian and clear cell renal carcinoma^14,19,21^. In vitro detection of GAGs in bladder cancer urine dates back to the early 1980s where Hennessey and Cutter described a potential relationship between increased GAG urine content and disease progression^22^. However, utility of urinary GAG analyses in bladder cancer has been limited partly due to the lack of appropriate methodology for detection of specific cancer-associated GAG subtypes. Several methods have been developed for the detection of GAGs in urine including precipitation with cationic dyes, electrophoresis, and capture and detection of specific structures in ELISA type assays^15,16,18,19,21^. Despite these efforts, cancer-specific GAG analyses of urine samples remain a technological challenge.

Bladder cancer has been reported to display changes in expression of GAGs and CSPGs in different stages of the disease. For example, high overall intratumor GAG content has been shown to correlate with bladder tumor grade and stage^23,24^. Some GAG subtypes such as ofCS are selectively expressed in malignancies, including bladder cancer^8,13^. Indeed, ofCS has been described to be highly expressed at various stages of bladder cancer where high ofCS levels correlate with resistance to chemotherapy and predicts poor survival of patients^13^. Also, CSPGs such as SDC1 and CSPG4 are highly expressed in bladder cancer and these CSPGs can indeed be modified with ofCS in a redundant manner, increasing the overall amount of ofCS in the tumors^13^. For those reasons, we decided to test whether rVAR2 could be used to detect cancer-derived ofCS in the urine of bladder cancer patients as an indication of disease. We thus developed a method to probe ofCS in urine from bladder cancer patients using a simple biochemical assay with rVAR2 as the detection reagent.

## Results

### Oncofetal chondroitin sulfate can be detected in urine from bladder cancer patients

**Fig. 1:**
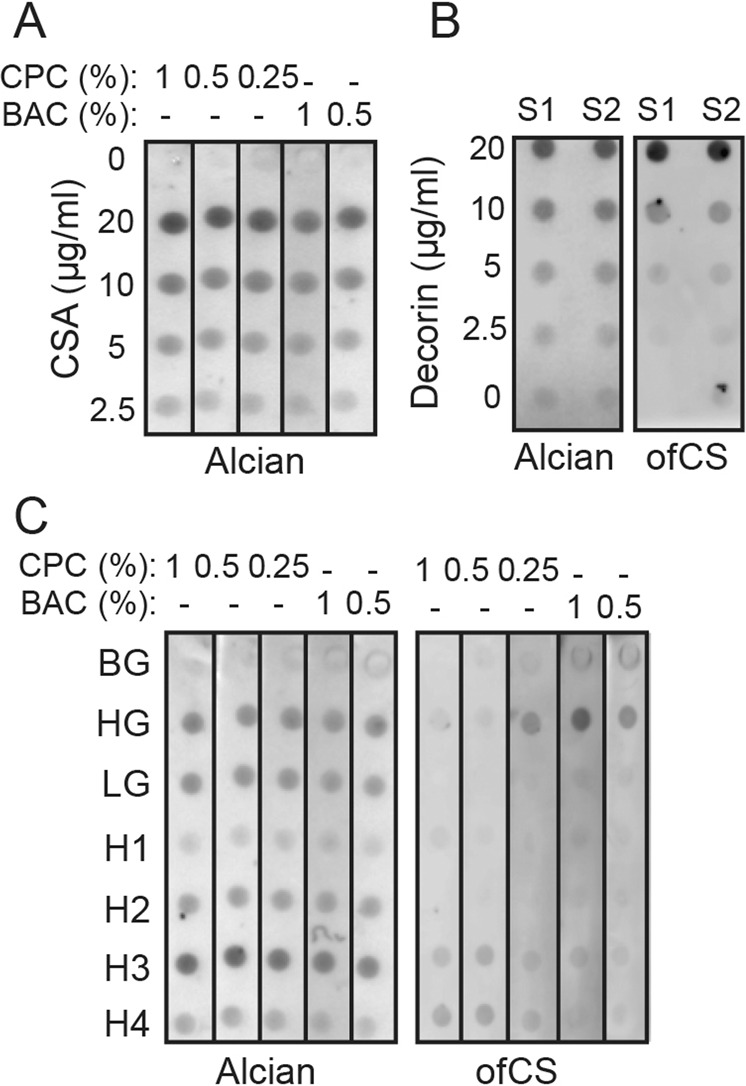
Soluble and protein bound ofCS detected by dot blot. **a** Different concentrations of CSA were dissolved in PBS and immobilized on a CPC or BAC derivatized membrane using a dot-blot apparatus following which, the membrane was stained with 10% (w/v) Alcian Blue. **b** Different concentrations of decorin were dissolved in PBS and tested similarly with rVAR2-HRP and Alcian Blue as described above. **c** Nitrocellulose membranes were derivatized using different concentration of either CPC vs. BAC and urine samples from cancer patients and healthy controls were applied to immobilize all the GAGs present in urine. The membranes were stained with rVAR2-HRP and visualized using enhanced chemiluminesence reagent. Following this, the membrane was stained with 10% (w/v) alcian blue. (CPC cetylpyridinium chloride, BAC benzalkonium chloride, BG background, HG high grade, LG low grade, H *h*ealthy individuals, S1 *r*eplicate1, S2 replicate 2).

For optimization of the assay, nitrocellulose membranes were treated with two different concentrations of cationic detergents, cetylpyridinium chloride (CPC) or benzyalkonium chloride (BAC). The treated membranes were then inserted into a dot-blot apparatus and a titration of chondroitin sulfate type A (CSA) in PBS was filtered through. Immobilization of CSA was observed with an Alcian Blue stain that stains most types of charged polysaccharides. We observed that both captured CS in a concentration-dependent manner as stained by Alcian Blue, but CPC facilitated marginally better binding to CS over BAC in this setting (Fig. 1a). While there is some CS present in the urine in free form, a large majority of the CS is attached to CSPG. To test whether rVAR2 could detect immobilized ofCSPGs from a liquid solution, we used purified bovine decorin, which contains CSA-modifications detectable by rVAR2^8^. In this analysis, we immobilized different concentrations of CSA-containing decorin in PBS onto BAC membranes and probed with rVAR2. Indeed, rVAR2 was able to detect CSA-modified decorin in a concentration-dependent manner (Fig. 1b). We then tested the system on urine from bladder cancer patients. Interestingly, when probing immobilized urine samples from high grade (HG) and low grade (LG) bladder cancer patients, as well as healthy individuals (H1-4) with rVAR2, strong reactivity was observed in the HG group (Fig. 1c). Also, rVAR2 binding to immobilized CSA was slightly better over background (BG) on BAC treated membranes as compared to membranes treated with CPC at different concentrations (Fig. 1c). Based on these results, treatment of the membranes with 0.5% BAC and blocking in 3% gelatin was used for all subsequent experiments. These data demonstrate that malarial rVAR2 protein can be used to probe for immobilized ofCS-modified proteoglycans in a BAC-primed dot-blot assay.

### A simple dot-blot assay for urinary oncofetal chondroitin sulfate analysis

**Fig. 2:**
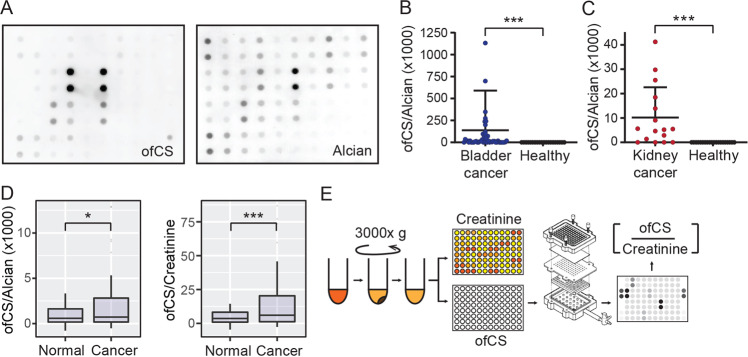
Bladder cancer detection based on ofCS expression in urine. **a** A full array of urine samples from the “training cohort” was immobilized on a BAC derivatized membrane and staining with rVAR2-HRP (ofCS) and Alcian Blue was carried out as described previously. Intensity of Alcian Blue staining was measured using the ImageStudio software. **b** Comparison ofCS readouts from patients with bladder cancer versus healthy individuals with ofCS expression readouts normalized to Alcian Blue. **c** Comparison ofCS readouts from patients with metastatic kidney cancer versus healthy individuals as in **b**. **d** Urinary creatinine in the samples was measured using a colorimetric assay kit. The difference in total glycan normalized ofCS expression between healthy and bladder cancer patients and difference in urinary creatinine normalized ofCS expression between healthy and bladder cancer patients was calculated on R. **e** Schematic representation of modified assay workflow. Boxes represent the 25th to 75th percentile with means at the 50th percentile and whiskers extend to the highest and lowest values within 1.5× of the upper and lower quartile distance with outliers shown as dots. Mann–Whitney test: *p < 0.05; **p < 0.01; ***p < 0.001.

We next tested the dot-blot assay on urine samples originating from a mixed cohort of bladder and prostate cancer patients and healthy controls. There was clear differential staining between rVAR2 and Alcian Blue in this cohort (Fig. 2a). Moreover, when focussing specifically on bladder cancer (Vancouver Cohort, Table 1) and using Alcian Blue as normalization, higher ofCS levels were observed in patients versus healthy individuals (Fig. 2b). Notably, we could also detect increased ofCSPG levels in a cohort of metastatic kidney cancer patients (Fig. 2c). We next tested whether urinary creatinine would perform better for normalization of the rVAR2-HRP signal as compared with Alcian Blue. The rationale for this was that urinary creatinine excretion rates are relatively constant amongst different individuals and within an individual over time, thereby providing a control for urine concentration independent of overall GAG concentration. Moreover, creatinine has been frequently used for normalizations in urine biomarker applications and its method of detection well established^25–27^. With urinary creatinine normalization, there was a better separation of cancer patients and healthy individuals as compared to Alcian Blue (Fig. 2d). We therefore concluded that normalization to urinary creatinine would be more accurate for clinical proof-of-concept analysis and we hence performed the remainder of the study as per outlined in the experimental workflow (Fig. 2e).

### Urinary oncofetal chondroitin sulfate reports on disease status pre and postsurgery

**Fig. 3:**
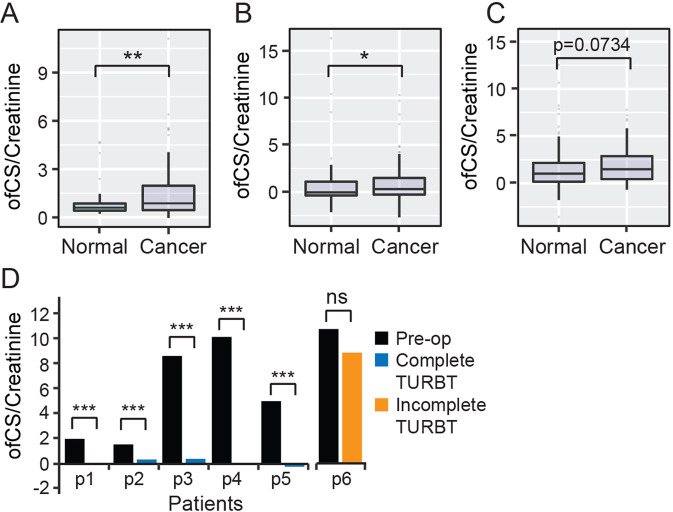
Validation of ofCS expression in different cohorts. **a** Expression analysis of ofCS between cancer-free individuals (normal) and bladder cancer patients in the UK cohort (*n* = 100) was carried out using the methods described previously and all data were analyzed using R. **b** Expression analysis of ofCS between cancer-free individuals and bladder cancer patients in the Spain cohort (*n* = 136). **c** Expression analysis of ofCS between cancer-free individuals and bladder cancer patients at the time of urine collection in the Tubingen Cohort (*n* = 198). **d** Urine samples of patients (*n* = 6) before and after treatment (tumor resection or cystectomy) were measured for ofCS expression and compared. Boxes represent the 25th to 75th percentile with means at the 50th percentile and whiskers extend to the highest and lowest values within 1.5× of the upper and lower quartile distance with outliers shown as dots. Mann–Whitney test: ***p < 0.001.

Using our rVAR2-based dot-blot assay (Fig. 2e), we next examined the feasibility of using ofCSPGs as a urinary biomarker for the detection of bladder cancer in three separate validation cohorts obtained from the Leeds Institute of Cancer and Pathology, the University of the Basque Country, Spain and from the University Hospital Tübingen. We observed a significant increase in the presence of urinary ofCS in patients with bladder cancer compared to healthy individuals and individuals who previously presented bladder cancer, but were disease-free at the time of urine collection in both the UK (Fig. 3a) and Spain (Fig. 3b) cohorts. We additionally observed a trend towards significance in the German cohort (Fig. 3c). Furthermore, we saw disappearance of urinary ofCS in 5/5 patients after trans-urothelial resection of bladder tumors (TURBT) or cystectomy, and persistent ofCS content in one patient with residual disease after TURBT (Fig. 3d).

### Urinary oncofetal chondroitin sulfate detection depends on tumor size and grade

**Fig. 4:**
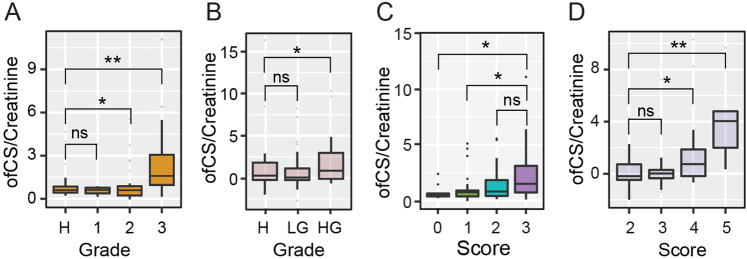
Urine ofCS expression is affected by both grade and size of tumor. **a** Urinary creatinine normalized expression of of CS expression based on tumor grade in patients from the UK cohort (*n* = 80) was analyzed on R. **b** Urinary creatinine normalized expression analysis of ofCS expression based on tumor grade in patients from the Spain cohort (*n* = 106). **c** Urinary creatinine normalized expression analysis of ofCS expression relative to tumor size in patients from the Spain cohort. (Score: 1—less than 2 cm; 2–2 cm to 4 cm; 3—more than 4 cm). **d** Comparison of urinary ofCS expression in Spain cohort patients relative to both size and grade. Boxes represent the 25th to 75th percentile with means at the 50th percentile and whiskers extend to the highest and lowest values within 1.5× of the upper and lower quartile distance with outliers shown as dots. (Scoring metric: low grade = 1; high grade = 2. Overall score = grade score + size score). (Legend: H—Cancer-free; LG—Low-grade; HG—High-grade). Mann–Whitney test: *p < 0.05; **p < 0.01; ***p < 0.001.

To further substantiate the data, we attempted to determine the correlation between tumor grade and the expression of urinary ofCS. In our first validation cohort (UK), we observed a significant increase in ofCS expression in patients with high-grade tumors only (Fig. 4a). The same pattern was observed in the second validation cohort (Spain) (Fig. 4b). Tumor size was available in the Spanish cohort, and we were able to demonstrate a significant correlation between tumor size and ofCS expression regardless of grade (Fig. 4c). Interestingly, there appeared to a bimodal distribution of ofCS expression across a number of different disease stages that we analyzed—particularly in the larger, more aggressive tumors. Finally, we did an aggregate score to reflect the contribution of size in the context of grade and repeated our analysis. The highest urinary ofCS levels were found in patients with large high-grade tumors (Fig. 4d).

## Discussion

A dot-blot assay developed by Karlsson et al., in which GAGs in urine were filtered through a membrane derivatized with a cationic detergent to allow for capture of polyanionic polysaccharides^28^. This assay allows for probing of specific GAG structures in the immobilized samples using lectins or structure specific antibodies^29^. As a further consideration, specific detection of GAG structures in immobilized samples has previously been shown to be dependent on the detergent used, despite sufficient immobilization^29^. We therefore reasoned that using a modified version of this assay, we should be able to probe urine samples for the presence of ofCS using rVAR2 given that rVAR2 specifically binds to ofCS^8^. Densitometry readings of the dot blot further allowed us to analyze the amount of ofCS present in the urine, thereby providing us with a quantitative measurement for the detection of urinary ofCS.

The results presented here suggest that urinary ofCS detection might be informative in the context of disease surveillance after TURBT or cystectomy and could potentially be a low-cost alternative to the current surveillance methods. While the UK and Spain cohorts exhibit a significant difference in ofCS expression between cancer-free and cancer-positive patients, this was not immediately apparent in the German cohort. We suspect that this minor deviation from significance could be due to the presence of outliers possibly attributed to differences in sample collection protocols, which were not standardized across the different cohorts. Furthermore, this cohort is biased in respect to tumor stage as a large number of tumors are of stage Ta. Since noninvasive, Ta tumors generally have low ofCS expression, this would likely be a confounding factor in particular cohort. The result from the German cohort becomes significant if a formal correction for outliers is performed.

Outliers as observed in the boxplot analyses could be attributed to other confounding variables that we were not able to account for in our cohorts, such as presence of additional urologic malignancies in individual patients, as well as differences in sample-to-sample liquid biopsy collection, and processing procedures. Collectively, our data signify that ofCS may be a candidate urinary biomarker for detection of bladder cancer.

Our data also demonstrate that ofCS is present in the urine of patients with bladder cancer in a grade- and tumor size-dependent manner. The finding that ofCS is also present in urine from kidney cancer patients suggests the possibility that changes in urine ofCSPG levels may be informative in additional cancer types. While highly intriguing, the clinical utility of ofCSPGs in urine remains to be explored. With disease-specific GAG binders such as rVAR2, we may be able to increase the resolution of GAG analyses in urine and develop informative noninvasive diagnostic, prognostic, or surveillance tools for future clinical use.

## Materials and methods

### Patient cohorts

The use of all patient samples was approved by the University of British Columbia’s Research Ethics Committee as the GU BioBank (approval number—H09-01628). Written consent was obtained from the patients. For the Vancouver discovery cohort, patients undergoing evaluation for initial presentation with symptoms of bladder cancer and those under surveillance for prior bladder cancer had their urine collected and banked. All collected samples were aliquoted and stored at −80 °C until needed for analysis. Our validation cohorts were obtained from the Leeds Institute of Cancer Studies, Pathology Universidad Pontificia de Salamanca and Eberhard–Karls University. The previously published cohort of metastatic renal cancer was provided by Dr. Umberto Basso (Istituto Oncologico Veneto IOV—IRCCS)^20^. An overview of the bladder cancer patient urine cohorts used in the validation is summarized in Table 1.

**Table 1:** Summary of clinical bladder cancer cohorts.

Characteristics	Discovery cohort		Validation cohort	Validation cohort	Validation cohort	
	Vancouver, Canada (*N* = 136)		Tübingen, Germany (*N* = 198)	Leeds, UK (*N* = 100)	Madrid, Spain (*N* = 136)	
Clinical context	Screening and surveillance		Surveillance	Surveillance	Screening and surveillance	
Bladder cancer status, *n* (%)						
Present (1)	66 (48.5%)		88 (44.4%)	80 (80%)	106 (77.9%)	
Absent (0)	70 (51.5%)		110 (55.6%)	20 (20%)	30 (22.1%)	
Clinical T stage, *n* (%)						
Tis	5 (7.6%)		5 (5.7%)	4 (5%)	0 (0)	
Ta	17 (25.8%)		54 (61.4%)	56 (70%)	39 (36.8%)	
I	13 (19.7%)		10 (11.4%)	9 (11.3%)	45 (42.4%)	
II	21 (31.8%)		13 (14.7%)	8 (10%)	19 (17.9%)	
III	6 (9.1%)		6 (6.8%)	2 (2.5%)	3 (2.8%)	
IV	5 (7.6%)		0 (0%)	1 (1.3%)	0 (0)	
Grade, *n* (%)			(^a^)			
I	LG	10 (15.1%)	23 (26.1%)	11 (13.8%)	LG	65 (61.3%)
II	HG	56 (84.9%)	30 (34.1%)	34 (42.5%)	HG	41 (38.7%)
III			30 (34.1%)	35 (46%)		

Summary of the cancer cohorts used in this study. The terms “screening” and “surveillance” signify the classification of disease-free individuals. In the surveillance cohorts, all patients classified as negative were disease-free at the urine collection but had a prior history of bladder cancer. In the screening and surveillance cohorts, patients classified as negative either had a prior history of bladder but were disease-free at the time of sample collection or had no prior history of bladder cancer but had other urologic pathologies.

^a^Specimens classified as Tis did not have a grade assigned.

### Dot blot

For dot--blot analysis, 1 mL aliquots of urine were thawed immediately before use and centrifuged at 3000 rpm to remove any cellular debris from the samples. The supernatant was then used for further downstream analysis. To begin the dot-blot assay, derivatization of 0.45 um PVDF membrane (Millipore Sigma, IPVH00010) was carried out by submersing the membrane into a 0.5% (w/v) benzalkonium chloride (Sigma, B-1383) in 30% 2-propanol solution. This was followed by four rounds of incubation and rinses with 150 mM NaCl solution to coat the membrane with an overall positive charge. The charged membrane was then placed inside a dot-blot apparatus and 20 uL per sample of urine samples were applied to the membrane. Subsequently, the membrane was blocked with 3% (w/v) gelatin for an hour followed by incubation with 4 µg/mL rVAR2 in 1.5% (w/v) gelatin for 1 h or overnight at 4 °C. The membranes were then washed and proved with a secondary HRP antibody targeting the V5 tag on rVAR2. Stained membranes were developed with SuperSignal™ West Femto Maximum Sensitivity Substrate (Thermo, 34095).

### Alcian blue staining

To carry out alcian blue staining, alcian blue was prepared by diluting a 20× alcian blue stock in 0.4 M GuHCl, 0.018 M Sulfuric Acid, 0.25% Triton-X. The membrane was then stained with the 1× alcian blue solution for 2 h at room temperature, following which, a 30 min wash step in 0.4 M GuHCl, 0.018 M Sulfuric Acid, 0.25% Triton-X was carried out. A final rinse with 150 mM sodium chloride was then carried out before analysis of the staining.

### Urinary creatinine measurements

All ofCS expression readouts (rVAR2-HRP) were normalized to urinary creatinine levels measured in the corresponding samples. Urinary creatinine was measured using the Creatinine (urinary) Colorimetric Assay Kit (Cayman Chemicals, 500701) according to manufacturer’s protocol.

### Statistical analyses

Graphing and statistical analyses were carried using the R software. The ofCS expression was statistically analyzed and significance, where indicated, was calculated using the Mann–Whitney test. Significance was determined as a *p*-value < 0.05. All analysis was carried out in a blinded manner wherein the clinical status of the patients was not revealed until after the analysis. No statistical method was used to predetermine patient sample size.

## Supplementary information

Supplementary Figure 1

Supplementary Figure 2
