# Supplementary figures and images for: A simple method for detecting oncofetal chondroitin sulfate glycosaminoglycans in bladder cancer urine
Source: Cell Death Discov. 2020 Jul 27;6:65. doi: 10.1038/s41420-020-00304-z (PMC7385127; doi:10.1038/s41420-020-00304-z)

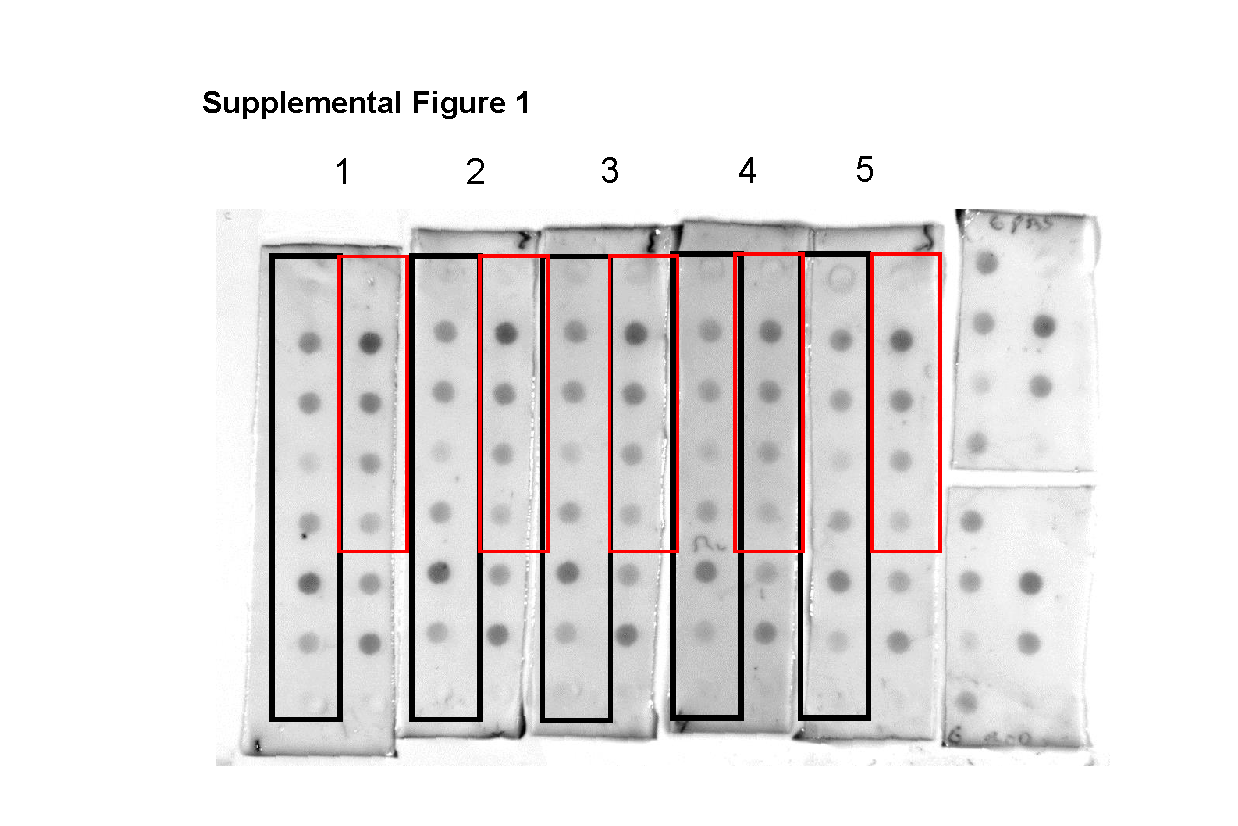

Supplement: Supplementary file 1 — Supplementary Figure 1 [file 41420_2020_304_MOESM1_ESM.png]

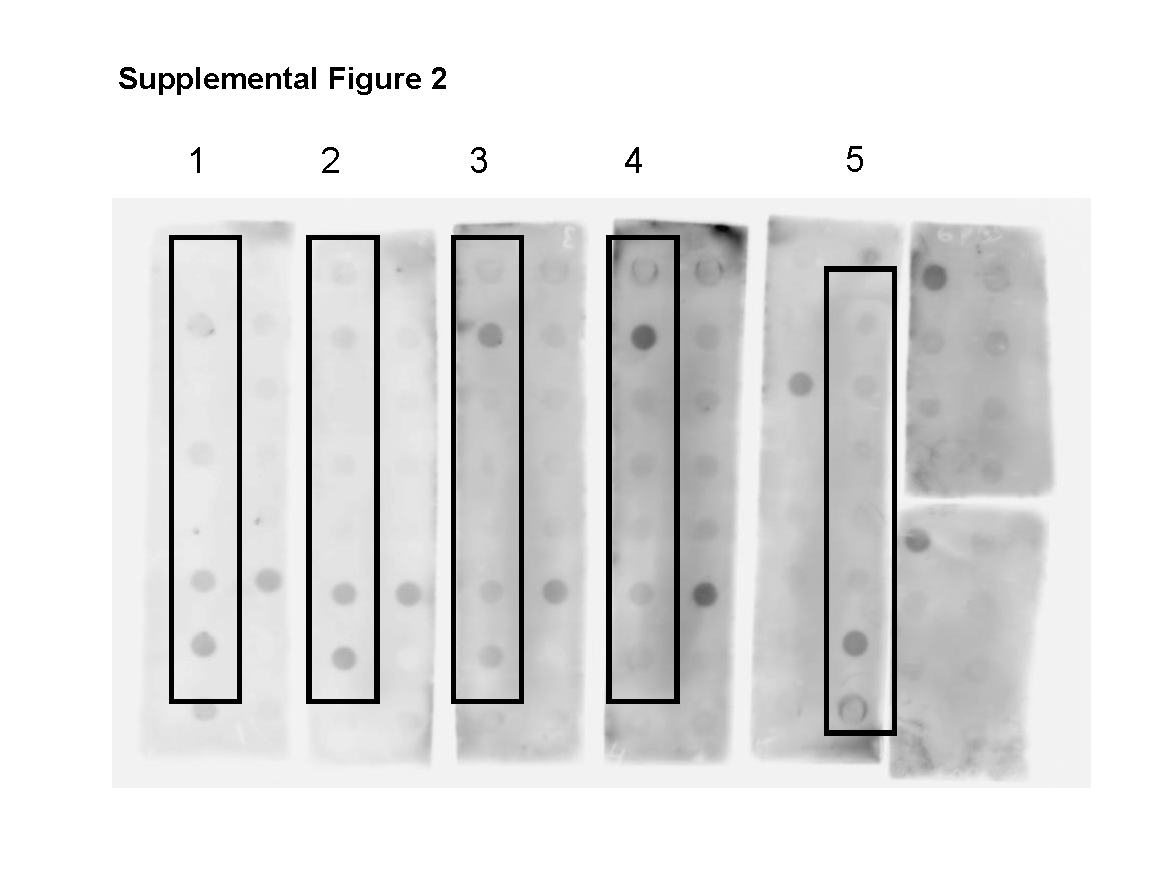

Supplement: Supplementary file 2 — Supplementary Figure 2 [file 41420_2020_304_MOESM2_ESM.png]
